# Chemical composition and *in vitro* evaluation of the cytotoxic and antioxidant activities of supercritical carbon dioxide extracts of pitaya (dragon fruit) peel

**DOI:** 10.1186/1752-153X-8-1

**Published:** 2014-01-03

**Authors:** Hui Luo, Yongqiang Cai, Zhijun Peng, Tao Liu, Shengjie Yang

**Affiliations:** 1 Guizhou Fruit Institute, Guizhou Academy of Agricultural Sciences, Guiyang 550006, P R China; 2 Research Institute of Traditional Chinese Medicine, Yangtze River Pharmaceutical Group Beijing Haiyan Pharmaceutical Co., Ltd, Beijing 102206, P R China; 3 State key Laboratory Breeding Base of Green Pesticide and Agricultural Bioengineering, Key Laboratory of Green Pesticide and Agricultural Bioengineering, Ministry of Education, Guizhou University, Guiyang 550025, P R China

## Abstract

**Background:**

*Hylocereus polyrhizus* and *Hylocereus undatus* are two varieties of the commonly called pitaya fruits, and pitaya fruits have gained popularity in many countries all over the world. However, studies on chemical composition and the nutritional quality of pitaya flesh peel are limited.

**Results:**

Extracts of pitaya (*H. polyrhizus* and *H. undatus*) peel were extracted by supercritical carbon dioxide extraction, and analyzed by gas chromatography–mass spectrometry analysis. Their cytotoxic and antioxidant activities were investigated. The main components of *H. polyrhizus* extract were *β*-amyrin (15.87%), *α*-amyrin (13.90%), octacosane (12.2%), *γ*-sitosterol (9.35%), octadecane (6.27%), 1-tetracosanol (5.19%), stigmast-4-en-3-one (4.65%), and campesterol (4.16%), whereas *H. undatus* were *β*-amyrin (23.39%), *γ*-sitosterol (19.32%), and octadecane (9.25%), heptacosane (5.52%), campesterol (5.27%), nonacosane (5.02%), and trichloroacetic acid, hexadecyl ester (5.21%). Both of the two extracts possessed good cytotoxic activities against PC3, Bcap-37, and MGC-803 cells (IC_50_ values ranging from 0.61 to 0.73 mg/mL), and the activities of their main components were also studied. Furthermore, these extracts also presented some radical scavenging activities, with IC_50_ values of 0.83 and 0.91 mg/mL, respectively.

**Conclusion:**

This paper provides evidence for studying the chemical composition of supercritical carbon dioxide extracts of pitaya peel and their biological activity.

## Background

Pitaya is often called “dragon fruit” following its bright red skin with green overlapping fins covering the fruit, which has gained popularity in many countries all over the world [1]. Three varieties that have been commercialized are *Hylocereus polyrhizus*, which has red-skinned fruit with red flesh, *Hylocereus undatus* (Red pitaya), which has red-skinned fruit with white flesh, and *Hylocereus megalanthus* (Yellow pitaya), which has yellow-skinned fruit with white flesh [2]. They belong to the vine cacti from the subfamily Cactoideae of the tribe Cacteae, and are native to the tropical forest regions of Mexico and Central and South America [3]. *H. polyrhizus* and *H. undatus* have recently drawn much attention from growers worldwide, because of their powerful antioxidative activity [4-6]. Betanin, phyllocactin, hylocerenin, and betacyanin with 5–*O*-glycosides or 6-*O*-glycosides have been discovered in many species of the Cactaceae family [7,8]. Furthermore, these types of compounds are responsible for many pharmacological activities such as antitumor, antioxidant, and anti-inflammatory actions. The objectives of this study were to evaluate the nutritional quality of pitaya flesh peel and study whether the peel of pitaya, the waste product from juice manufacture, could be utilized as a potential alternative for various sources of nutrients or antioxidants to improve human health.

The aim of the present study was, as a first step, to determine the chemical composition of supercritical carbon dioxide extracts of the peel of pitaya (*H. polyrhizus* and *H. undatus*) by gas chromatography–mass spectrometry (GC-MS) analysis. In a second step, we examined the *in vitro* cytotoxic and antioxidant activities of the two extracts by MTT and DPPH assays, respectively. We compared their activities with the activity of the major component of each extract sample. To our knowledge, there are no published reports on the chemical compositions, cytotoxic and antioxidant activities of supercritical carbon dioxide extracts of the pitaya (*H. polyrhizus* and *H. undatus*) peel.

## Results and discussions

### Chemical composition of supercritical carbon dioxide extract

The pale yellowish extracts of the peel of *H. polyrhizus* and *H. undatus* were obtained by supercritical carbon dioxide extraction, with yield of 1.81% and 1.25%, respectively. These extracts were analyzed by GC-MS. The identified constituents of the two extracts and their retention time are shown in Table 1. All the compounds are arranged in the order of their elution from the HP5-MS column.

**Table 1:** Chemical composition of supercritical carbon dioxide extracts of pitaya peel

**No.**	**Compounds**	**RT**^**a**^	***H. polyrhizus***	***H. undatus***
			**Area (%)**	
1	*n*-Hexadecanoic acid	39.85	1.46	2.37
2	1-Hexadexyne	44.47	-	0.68
3	(*Z*, *Z*)-9, 12-Octadecadienoic acid	44.97	-	1.62
4	2-Chloroethyl linoleate	45.01	0.38	-
5	Oleic acid	45.19	1.17	1.25
6	Octacosane	49.61	12.2	-
7	17-Pentatriacontene	50.83	1.15	-
8	Trichloroacetic acid, hexadecyl ester	54.71	-	5.21
9	1-Nonadecene	54.79	3.17	-
10	6-Tetradecanesulfonic acid, butyl ester	55.81	-	1.25
11	1,2-Benzenedicarboxylic acid, mono (2-ethylhexyl) ester	56.11	0.31	0.81
12	Phthalic acid, 6-ethyloct-3-yl 2-ethylhexyl ester	56.19	1.21	-
13	Eicosane	59.44	3.64	1.92
14	Tetratriacontane	59.51	1.04	-
15	1-Tetracosanol	59.80	5.19	-
16	Heptacosane	59.93	0.44	5.52
17	Campesterol	60.97	4.16	5.27
18	Stigmasterol	62.59	1.21	2.75
19	Squalene	62.86	0.22	0.64
20	11-Hexacosyne	63.07	1.24	-
21	Octadecanal	63.18	-	3.55
22	Nonacosane	64.52	1.43	5.02
23	Octadecane	64.87	6.27	9.25
24	*γ*-Sitosterol	65.12	9.35	19.32
25	*α*-Amyrin	66.15	13.90	-
26	Hexadecyl oxirane	67.55	-	1.54
27	*β*-Amyrin	67.81	15.87	23.39
28	Ergosta-4,6,8(14),22-tetraen-3-one	68.24	-	1.46
29	Docosane	68.82	3.19	-
30	Stigmast-4-en-3-one	69.83	4. 65	-
31	*β*-Sitosterol	72.84	2.46	-

Note: Relative proportions of the extract constitutes were expressed as percentages.

^a^Retention time.

**Figure 1:**
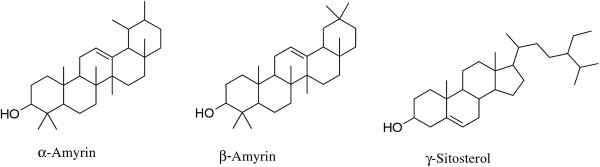
The structures of the main components of pitaya peel extracts.

Regarding the chemical composition of the two extracts tested, they were shown to be complex mixtures of many components. Table 1 shows the identified compounds (in total, 31 compounds), retention time and percentage obtained by GC-MS. And the molecular structures for the main compounds of these extracts are shown in Figure 1.

A total of 24 components in *H. polyrhizus* extract, representing 90.66% of the total composition, were identified, of which 29.77% were triterpenoids and 16.46% were steroids. Its extract was characterized by a high content of *β*-amyrin (15.87%), *α*-amyrin (13.90%), octacosane (12.2%), *γ*-sitosterol (9.35%), octadecane (6.27%), 1-tetracosanol (5.19%), stigmast-4-en-3-one (4.65%), and campesterol (4.16%).

The predominant constituents of *H. undatus* extract were *β*-amyrin (23.39%), *γ*-sitosterol (19.32%), and octadecane (9.25%), which formed approximately a half of the extract. Heptacosane (5.52%), campesterol (5.27%), nonacosane (5.02%), and trichloroacetic acid, hexadecyl ester (5.21%) were also present at significant concentration. A total of 19 components were identified, comprising 92.82% of the total extract. Moreover, its extract was also dominated by triterpenoids (23.39%) and steroids (19.32%).

In conclusion, both of *H. polyrhizus* and *H. undatus* contained mostly triterpenoids and steroids. In contrast, the content of triterpenoids in supercritical carbon dioxide extract of *H. polyrhizus* was higher than that of *H. undatus*, whereas the extract of *H. undatus* had higher content of steroids. It would also be worth pointing out that the constituents of the two extracts are normally influenced by several factors such as geographical, climatic, seasonal and experimental conditions.

### Cytotoxic activity

**Figure 2:**
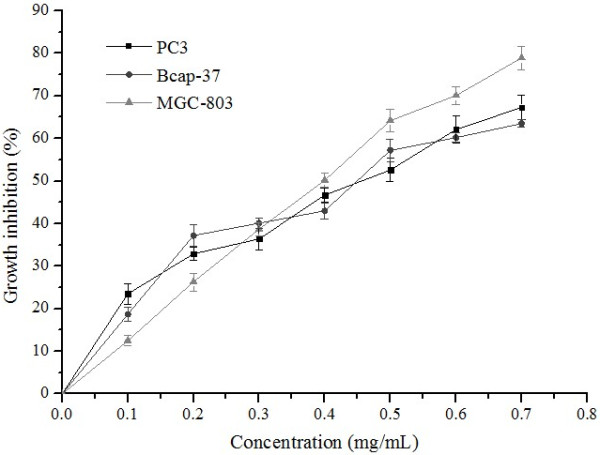
**Effect of *H. polyrhizus *** **extract on proliferation of cancer cells.**

**Figure 3:**
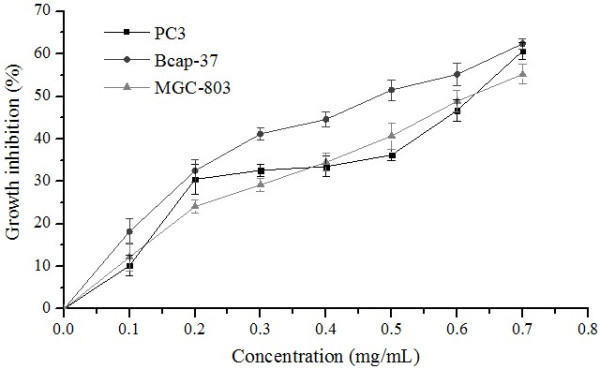
**Effect of *H. undatus *** **extract on proliferation of cancer cells.**

To determine the cytotoxic activity of supercritical carbon dioxide extracts of pitaya (*H. polyrhizus* and *H. undatus*) peel against cancer cell lines PC3 (human prostate cancer cell line), Bcap-37 (human breast cancer cell line), and MGC-803 (human gastric cancer cell line), cytotoxicity MTT assay was carried out, and net growth inhibition was calculated comparing to a negative control growth. Adriamycin (ADM) was used as a positive control. The inhibitory ratios of ADM after 72 h of treatment at 0.1 mg/mL against the three cell lines were 97.2%, 99.3%, and 98.1%. Of all extracts tested at maximum concentration (0.7 mg/mL), the inhibitory ratios of *H. polyrhizus* and *H. undatus* extracts were 67.3% and 60.7% against PC3 cells, 63.5% and 62.4% against Bcap-37 cells, and 78.9% and 55.2% against MGC-803 cells, respectively. Further experiments found that proliferation of these three cells were significantly inhibited by these extracts in a concentration-dependent manner, as shown in Figures 2 and 3. The IC_50_ values of *H. polyrhizus* extract on these three cells were 0.61, 0.45, and 0.43 mg/mL, respectively, while for *H. undatus* extract, the IC_50_ values were 0.64, 0.47, and 0.73 mg/mL, respectively. Thus it can be seen that the inhibitory effect on cancer cells of *H. polyrhizus* was stronger than that of *H. undatus*, especially on MGC-803 cells.

Steroids and pentacyclic triterpenoids are the most important classes of natural products occurring widely in the plant kingdom [9,10]. They have been shown to possess several medicinal properties including anticancer and anti-HIV activities [11]. Thao *et al.* found that *β*-amyrin exhibited some cytotoxicity against A549 and HL-60 cancer cell lines with IC_50_ values of 46.2 and 38.6 μM, respectively [12]. In 2012, Lin *et al.* studied the chemical constituents of *Rabdosia serra* (MAXIM.) HARA, and found *β*-sitosterol isolated from the plant have significant cytotoxic activities against HepG-2, MCF-7, and HL-60 cells [13]. Stigmast-4-en-3-one also displayed high antitumor-promoting activity [14]. Thus, to determine whether these compounds were responsible for the activities of these extracts, we evaluated the cytotoxic activities of these compounds against PC3, Bcap-37, and MGC-803 cells. The results are shown in Table 2.

**Table 2:** **Effect of steroids and triterpenoids from supercritical carbon dioxide extracts of *H. polyrhizus *** **and *H. undatus *** **against cell viability of different cancer cell lines**

**Compound**	**IC**_**50 **_**(μM)**^**a**^		
	**PC3**	**Bcap-37**	**MGC-803**
*α*-Amyrin	>100^b^	>100	>100
*β*-Amyrin	73.2 ± 1.02	78.4 ± 0.93	51.9 ± 0.87
*β*-Sitosterol	74.4 ± 0.65	58.2 ± 0.44	43.8 ± 0.63
Stigmast-4-en-3-one	65.4 ± 1.13	79.3 ± 0.49	56.9 ± 0.81
ADM^c^	1.09 ± 0.18	1.34 ± 0.30	0.83 ± 0.22

^a^Agent concentration (μM) that inhibited cell growth by 50% at 72 h after treatment.

^b^When 50% inhibition could not reached at the highest concentration, then >100 μM was given.

^c^Adriamycin, positive control.

It can be seen from the IC_50_ values that *β-*amyrin, *β*-sitosterol, and stigmast-4-en-3-one suppressed proliferation of the above three cancer cell lines in different extents (IC_50_ values of 43.8-79.3 μM). These compounds showed similar inhibition activity against PC3 and MGC-803 cells, while the proliferation inhibition of MGC-803 cells was superior to other kinds of cancer cells. However, *α*-amyrin displayed weak activities against the three cells. These finding indicated that *β*-amyrin, *β*-sitosterol, and stigmast-4-en-3-one may be responsible for the activities of the two extracts.

### Antioxidant activity

**Figure 4:**
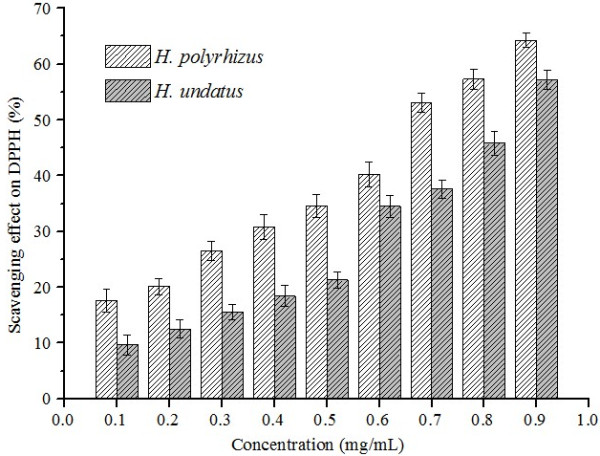
Free radical scavenging properties of pitaya peel extracts.

The principle of *in vitro* antioxidant activity is based on the availability of electrons to neutralize an free radicals [15,16]. In this study, the antioxidant activities of supercritical carbon dioxide extracts of *H. polyrhizus* and *H. undatus* were evaluated by DPPH radical scavenging assay, with vitamin C (Vc) as the positive control. And the negative control group was treated with ethanol. The two extracts and Vc were dissolved in ethanol. Each experiment was repeated at least three times. The scavenging rate of Vc at 0.1 mg/mL was 98.9%. DPPH free-radical scavenging properties of the two extracts are present in Figure 4. A lower IC_50_ value and greater DPPH radical scavenging percentages indicate higher antioxidant activity. Both of the two extracts exhibited some antioxidant activities. The IC_50_ values of *H. polyrhizus* and *H. undatus* extracts were 0.83 and 0.91 mg/mL, respectively. It also can be seen from Figure 4 that the two extracts showed dose dependent antioxidant activity.

Antioxidants terminate these chain reactions by removing free radical intermediates, and inhibit other oxidation reactions, and they do this by being oxidized themselves [17-19]. High phenolic content were usually correlated with high radical scavenging activity [20]. Choo *et al.* found that *H. polyrhizus* and *H. undatus* had great antioxidant properties, because of high content of polyphenols [2]. Moreover, polyphenols can be extracted by supercritical carbon dioxide extraction [21]. Hence, antioxidant activities of the pitaya peel extracts were most probably due to the presence of polyphenols, which have the hydrogen-donor ability to scavenge the free radicals. However, the polyphenols were not detected by GC-MS. Studies of the content of polyphenols in the extracts are currently underway.

## Conclusions

In summary, the composition of supercritical carbon dioxide extracts of pitaya (*H. polyrhizus* and *H. undatus*) peel has been analyzed by GC-MS, and their cytotoxic and antioxidant activity were investigated. The predominant constituents of *H. polyrhizus* extract were *β*-amyrin (15.87%), *α*-amyrin (13.90%), octacosane (12.2%), *γ*-sitosterol (9.35%), whereas *H. undatus* were *β*-amyrin (23.39%), *γ*-sitosterol (19.32%), and octadecane (9.25%). The two extracts showed a wild range of cytotoxic activities against PC3, Bcap-37, and MGC-803 cells, and it was found that *β*-amyrin, *β*-sitosterol, and stigmast-4-en-3-one, the main components, were responsible for their activities. In addition, they had some DPPH radical scavenging activities, with IC_50_ values of 0.83 and 0.91 mg/mL, respectively.

There is a trend to find cytotoxic and antioxidant materials from natural products in the modern medical industry. The above results show that supercritical carbon dioxide extracts of pitaya (*H. polyrhizus* and *H. undatus*) peel could be a potential source of compounds with cytotoxic and antioxidant activities and the results provide a reference point for further research on the chemical components of supercritical carbon dioxide extracts of pitaya peel as well as for their utilization.

## Materials and methods

### General procedures and reagents

The melting points of the products were determined using an XT-4 binocular microscope (Beijing Tech Instrument Co. Ltd., Beijing, China). ^13^C NMR were recorded using a JEOL-ECX500 spectrometer at 22°C, with tetramethylsilane as the internal standard and CDCl_3_ as the solvent. Column chromatography was performed using silica gel (200–300 meshes) (Qingdao Marine Chemistry Co., Qingdao, China) and Sephadex LH-20 (GE Healthcare Bio-Sciences AB, Uppsala, Sweden). Sodium dodecyl sulfate (SDS) were purchased from Beijing Dingguo CO., Ltd; 2,2-diphenlyl-1-picrylhydrazyl (DPPH) and vitamin C (Vc) were purchased from Aladdin Reagent Inc; 3-(4,5-Dimethylthiazol-2-yl)-2,5-diphenyl tetrazolium bromide (MTT) and DMSO were purchased from Roche Molecular Biochemicals (1465–007); Adriamycin (ADM) was purchased from Zhejiang Hisun Pharmaceutical Co., Ltd; *α*-amyrin, *β*-sitosterol, and stigmast-4-en-3-one had been prepared in previous work [22,23]. *β*-amyrin was isolated from supercritical carbon dioxide extract of *H. undatus* peel, and its purification process and NMR data are presented in Additional file 1. All other chemicals were of analytical reagent grade and used without further purification.

### Plant materials

Fresh peel of pitaya (*H. polyrhizus* and *H. undatus*) were collected from Guiyang, Guizhou province in China, in July 2013. Voucher specimens were deposited at Guizhou Fruit Institute, Guiyang, China.

### Supercritical carbon dioxide extraction

About 250 g of dried peel of pitaya (*H. polyrhizus* and *H. undatus*) were cut into pieces and submitted to extraction. A CO_2_ flow rate of 30 L/h and an extraction period of 60 min were used. The extraction was performed under a pressure of 30 MPa and at a temperature of 40°C. The two extracts obtained by supercritical carbon dioxide extraction assay were pale yellowish. These extracts were dried over anhydrous Na_2_SO_4_ and placed at a low temperature in the refrigerator until analysis.

### Gas chromatography-mass spectroscopy (GC-MS) analysis

A gas chromatographic-mass spectral analysis was performed on the extracts using an Agilent 6890 GC with Agilent 5973 mass selective detector (EI-MS, electron energy = 70 eV, scan range = 10-550 amu), and a fused silica capillary column (HP-5 ms, 30 m × 0.25 mm) coated with 5% phenyl methyl siloxane (0.25 μm phase thickness). The carrier gas was helium (99.999%) with a flow rate of 1.0 mL/min. The injector temperature was 250°C, and the oven temperature was programmed to 50°C for 2 min, and then increased to 290°C at a rate of 5°C/min. The interface temperature was 280°C. A 1% (*w/v*) solution of each sample in dichloromethane CH_2_Cl_2_ was prepared, and 1 μL was injected using a split injection technique with split ratio 20:1. The components were identified by comparison of their mass spectra with those of the NIST 5 mass spectra library.

### Cell lines and culture

PC3, Bcap-37, and MGC-803 cell lines were obtained from the Cell Bank of the Chinese Academy of Sciences (Shanghai, China). The entire cancer cell lines were maintained in the RPMI 1640 medium. They were supplemented with 10% heat-inactivated fetal bovine serum (FBS). All cell lines were maintained at 37°C in a humidified 5% carbon dioxide and 95% air incubator.

### MTT assay

All the extracts or compounds were dissolved in DMSO and subsequently diluted in the culture medium before treatment of the cultured cells. When PC3, Bcap-37, and MGC-803 cells were 80-90% confluent, they were harvested by treatment with a solution containing 0.25% trypsin, thoroughly washed and resuspended in supplemented growth medium. Cells were plated in 100 μL of medium/well (2 × 10^3^/well) in 96-well plate. After incubations overnight, the cells were treated with extracts or compounds in RPMI 1640 with 10% FBS for 72 h. In parallel, the cells treated with 0.1% DMSO served as negative control and ADM as positive control. After 72 h, 100 μL of MTT was added, and the cells were incubated for 4 h. The MTT-formazan formed by metabolically viable cells was dissolved in 100 μL of SDS for 12 h. The absorbance was then measured at 595 nm with a microplate reader (BIO-RAD, model 680), which is directly proportional to the number of living cells in culture [24-26]. The percentage cytotoxicity was calculated using the formula.

$$\begin{array}{ll} \%\;\mathrm{Cytotoxicity} & =\left[\left({\mathrm{Control}}_{\mathrm{abs}}-{\mathrm{Blank}}_{\mathrm{abs}}\right)\right)\\ & \;\left(-\left({\mathrm{Test}}_{\mathrm{abs}}-{\mathrm{Blank}}_{\mathrm{abs}}\right)\right]\\ & \;/\left({\mathrm{Control}}_{\mathrm{abs}}-{\mathrm{Blank}}_{\mathrm{abs}}\right)\times 100 \end{array}$$

### DPPH free radical scavenging assay

The DPPH free radical scavenging assay has been widely used to evaluate the antioxidant capacity, which is stable due to its resonance stability and special blockade of benzene rings [27,28]. The purple chromogen radical DPPH is reduced by antioxidant compounds to the corresponding pale yellow hydrazine [29]. The antioxidant activity of plant extracts and antioxidant standard were evaluated on the basis of radical scavenging effect of the stable DPPH free radical. In its radical form, DPPH has a characteristic absorption at 515 nm in ethanol, which disappears with acceptance of an electron from the antioxidant sample. All tested samples were dissolved in ethanol. 100 μL of DPPH in ethanol was added into a 96-well plate, and was mixed with the test samples (100 μL) at different concentrations. After shaken for 60 s in microplate reader, it was left in the dark at 37°C for 30 min. The absorbance was then measured at 515 nm with a microplate reader (BIO-RAD, model 680). All experiments were carried out in triplicate. Ethanol was used as the blank control and vitamin C served as positive control [30,31]. The DPPH radical scavenging activity of the extracts or compounds were calculated according to the following formula.

$$\begin{array}{ll} \%\;{\mathrm{DPPH}}_{\mathrm{scavenging}\;\mathrm{activity}} & =\left({\mathrm{OD}}_{\mathrm{blank}}-{\mathrm{OD}}_{\mathrm{sample}}\right)/{\mathrm{OD}}_{\mathrm{blank}}\\ & \;\times 100 \end{array}$$

### Statistical analysis

All statistical analyses were performed using SPSS 10.0, and the data were analyzed using one-way ANOVA. The mean separations were performed using the least significant difference method. Each experiment was performed in triplicate, and all experiments were run thrice and yielded similar results. Measurements from all the replicates were combined, and the treatment effects were analyzed.

## Competing interests

The authors declare that they have no competing interest.

## Authors’ contributions

HL and YC collected and identified the plant material, and drafted the manuscript. ZP performed the GC-MS analysis, identified the components and drafted the manuscript. TL took part of the bioassay experiments. SY identified the components and took part of the bioassay experiments. All authors read and approved the final manuscript.

## Supplementary Material

Additional file 1 **Experimental details and data of *β*-amyrin.** Which includes the experimental procedure, spectroscopic data, and copies of ^1^H NMR and ^13^C NMR of *β*-amyrin. Click here for file
