# Supplementary material for: Chemical composition and in vitro evaluation of the cytotoxic and antioxidant activities of supercritical carbon dioxide extracts of pitaya (dragon fruit) peel
Source: Chem Cent J. 2014 Jan 3;8:1. doi: 10.1186/1752-153X-8-1 (PMC3880984; doi:10.1186/1752-153X-8-1)
Supplement: Additional file 1 — Experimental details and data of β -amyrin. Which includes the experimental procedure, spectroscopic data, and copies of 1H NMR and 13C NMR of β-amyrin. [file 1752-153X-8-1-S1.doc]

Additional file 1

# Chemical composition and *in vitro* evaluation of the cytotoxic and antioxidant activities of supercritical carbon dioxide extracts of pitaya (dragon fruit) peel

Hui Luo^1,3¶^, Yongqiang Cai^1¶^, Zhijun Peng^1^, Tao Liu^1^, and Shengjie Yang^2,3*^

^1^ Guizhou Fruit Institute, Guizhou Academy of Agricultural Sciences, Guiyang 550006, P.R. China

^2^ Research Institute of Traditional Chinese Medicine, Yangtze River Pharmaceutical Group Beijing Haiyan Pharmaceutical Co., Ltd, Beijing 102206, PR China

^3^State key Laboratory Breeding Base of Green Pesticide and Agricultural Bioengineering, Key Laboratory of Green Pesticide and Agricultural Bioengineering, Ministry of Education, Guizhou University, Guiyang 550025, PR China

^¶^ Both authors contributed equally to this work.

^*^Corresponding author. Tel: +86(10)8072-8999-6259; Fax: +86(10)8072-8999-6251; E-mail address: yangsj2003@gmail.com.

Email addresses:

Hui Luo: luohui8732@163.com

Yongqiang Cai: caiyongqiang08@126.com

Zhijun Peng: [zhijunpeng1980@126.com](mailto:zhijunpeng1980@126.com)

Tao Liu: 603913045@qq.com

Shengjie Yang^*^: yangsj2003@gmail.com.

**Extraction and isolation**

The supercritical carbon dioxide extract of *H. undatus* peel was subjected to CC on silica gel (200-300 mesh) eluted with a gradient of petroleum ether-EtOAc (20/1, 10/1, 5/1, 2/1 and 1/1, *v/v*) to get 5 fractions, namely, fractions 1-5. Fraction 3 was applied to a silica gel (200-300 mesh) column eluted with petroleum ether-EtOAc (10:1 to 1:1) to yield *β*-amyrin (17 mg).

*β*-Amyrin, white powder; mp 192-194^o^C; ESI-MS *m/z*:426 [M-H]^-^; ^13^C NMR (125 MHz, CDCl_3_) δ: 144.8 (C-13), 122.6 (C-12), 79.1 (C-3), 46.7 (C-18), 46.2 (C-19), 54.6 (C-5), 47.2 (C-9), 41.2 (C-14), 39.3 (C-8), 38.2 (C-22), 38.1 (C-1), 37.8 (C-4), 36.8 (C-10), 32.9 (C-29), 32.1 (C-17), 31.9 (C-7), 31.7 (C-20), 27.3 (C-28), 26.0 (C-16), 26.0 (C-27), 24.6 (C-21), 23.5 (C-11),23.2 (C-30), 18.4 (C-6), 17.0 (C-26), 15.5 (C-24), 15.3 (C-25). The above data were identical to the literature data [1].

**References**

1. Wang H, Zhang X, Pan L, Yang S, Ma Y, Luo X: **Chemical constituents from Euphorbia wallichii.** *Nat Prod Res Dev* 2003, **15**: 483-386.
